# Surgical portfolios: A systematic scoping review

**DOI:** 10.1016/j.sipas.2022.100107

**Published:** 2022-07-06

**Authors:** Eleanor Jia Xin Chong, Ming Jia Wang, Jia Yin Lim, Grace Shen Shen, Misha Jing Yi Chow, Kai Kee Koh, Annabelle Jia Sing Lim, Daniel Zhihao Hong, Jacquelin Jia Qi Ting, Anushka Pisupati, Betrand Kai Yang Lam, Yun Ting Ong, Min Chiam, Stephen Mason, Lalit Kumar Radha Krishna, Si Ying Tan

**Affiliations:** a Yong Loo Lin School of Medicine, National University of Singapore, NUHS Tower Block, 1E Kent Ridge Road, Level 11, 119228, Singapore; b Division of Supportive and Palliative Care, National Cancer Centre Singapore, 11 Hospital Dr, 169610 Singapore; c Division of Cancer Education, National Cancer Centre Singapore, 11 Hospital Dr, 169610 Singapore; d Palliative Care Unit, Academic Palliative & End of Life Care Centre, University of Liverpool, UK; e Duke-NUS Medical School, 8 College Rd, 169857 Singapore; f Centre of Biomedical Ethics, National University of Singapore, 21 Lower Kent Ridge Rd, Singapore 119077; g PalC, The Palliative Care Centre for Excellence in Research and Education @ Dover Park Hospice, 10 Jalan Tan Tock Seng, 308436 Singapore; h SingHealth Duke-NUS Breast Centre, Singapore General Hospital, Outram Road 169608 Singapore

**Keywords:** Surgical portfolio, Medical education, Teaching, Assessment, Reflection, ACGME competencies, Practice-based learning and improvement (PBLI), Professionalism

## Abstract

**Introduction:**

The need for longitudinal and comprehensive evidenced development of surgical knowledge, skills and attitudes, and the attainment of appropriate competencies have spotlighted portfolio use. Yet variations in structure, content and roles as well as its resource-heavy reputation have limited its impact. The purpose of this study is to map prevailing data on the use of portfolios in postgraduate surgical education to address a lack of e-portfolio use and guide its design and structuring in surgery.

**Material and methods:**

A Systematic Evidenced Based Approach (SEBA) guided systematic scoping review (SSR) (SSR in SEBA) was conducted to map prevailing data. The constructivist ontological approach used allowed for enhanced reflexivity in the analysis and discussion.

**Results:**

13,092 articles were reviewed, 839 full-text articles were evaluated, and 37 articles were included. Concurrent thematic and content analysis revealed similar themes and categories which were the role of portfolios, evaluation of portfolio use, and implementation of e-portfolios.

**Discussion:**

Lessons from traditional surgical portfolios can be employed to enhance the design, support and utility of e-portfolios and yet maintain as a balanced trainee-led perspective of their progress. Focused on documenting the trainee's competencies, achievements, experiences, feedback, and reflections, a variety of methods are presently used to assess the reliability, validity, educational impact, acceptability and feasibility of surgical portfolios. The use of e-portfolios, in particular, has been adopted due to its accessibility, portability and convenience. Beyond its flexibility in access, repository and content, e-portfolios offer increased user accessibility, better quality interactions and smoother facilitation, clarification, and feedback.

**Conclusions:**

The data in this SSR in SEBA underscores the need for systematic assessment tools to evaluate e-portfolio as well as evaluate the programs as a whole. Further research into training mentors to support e-portfolio use, assess progress and ensure effective oversight of e-portfolios are required.

## Highlights

• The ethos of portfolios should be seen to as a tool for development rather than simple for the assessment of progress.
• Portfolios are a powerful tool in surgical education as they foster continuous personal and professional growth by capturing the postgraduate medical student's development of longitudinal surgical competencies and enhancing their self-directed learning.
• Beyond its flexibility in access, repository and content, e-portfolios offer increased user accessibility, better quality interactions and smoother facilitation, clarification, and feedback.

## Introduction

Postgraduate surgical training is evolving, shifting from a syllabus-based content-driven system to a longitudinal competency based process [1], [2], [3], [4]. The Royal College of Surgeons [1,2] and Accreditation Council for Graduate Medical Education (ACGME) [3], [4], [5], [6], [7], also expect surgical trainees to evidence appropriate levels of professionalism; effective communication, team working, decision making and leadership skills [8], [9], [10]; and exhibit appropriate situational awareness [11]. Such knowledge, skills and attitudes inculcated over the course of their training and evidenced through mix of longitudinal assessments [12], reflections [13] and feedback [14] have brought portfolios to the fore in current thinking [15,16].

Seen as a “collection of products prepared by the resident that provides evidence of learning and achievement related to a learning plan” that has found traction amongst surgical training programmes such as the Irish Intercollegiate Surgical Curriculum Programme (ISCP) [17] and the ACGME Toolbox of Assessment Methods [15], portfolios continue to suffer from a lack of consistency in their structure raising questions as to their feasibility and efficacy(12–14, 17)_ENREF_13_ENREF_14_ENREF_15. Concurrent calls for portability, accessibility and flexibility in present formulations have focused attention on the employ of e-portfolios which is quite simply seen as “*the combination of portfolio and technology*” [2,10,16,17]_ENREF_15_ENREF_17. E-portfolios in surgical training are also seen to meet the need for a longitudinal perspective of progress, promote timely feedback and potentially reduce the time and resource burden upon users and administrators [2,10,17]_ENREF_15.

To contend with a relative dearth of data that has compromised attempts to facilitate surgical e-portfolio use, this review seeks to understand the role, content and structure of current accounts of portfolios in surgical education, and explore how best to integrate these findings to improve e-portfolios in surgical training.

## Materials and methods

**Fig. 1:**
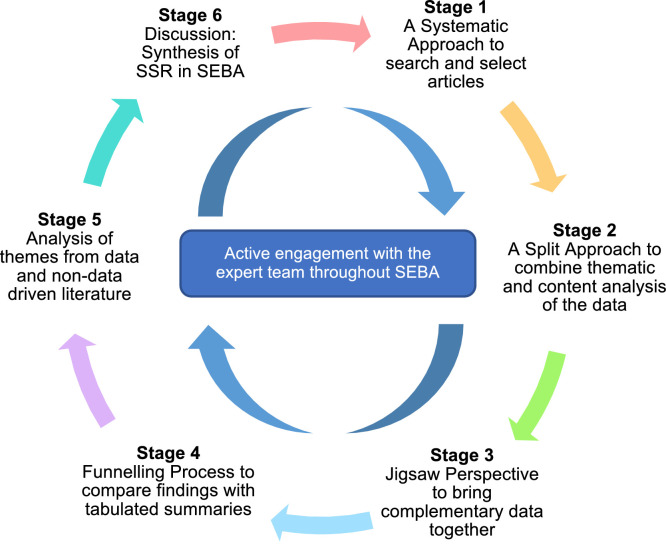
The SEBA Process.

A Systematic Evidenced Based Approach guided systematic scoping review (henceforth SSR in SEBA) is used to map current use of postgraduate surgical portfolios [18], [19], [20], [21]. This SSR in SEBA is overseen by an expert team comprised of medical librarians and local educational experts and clinicians who guide, oversee and support all stages of SEBA to enhance the reproducibility and accountability of the process [18,[22], [23], [24], [25], [26], [27], [28], [29], [30], [31], [32], [33], [34], [35]]. This SSR in SEBA is also shaped by SEBA's constructivist ontological perspective and relativist lens as well as the principles of interpretivist analysis to enhance reflexivity of the research, analysis and discussions [36], [37], [38], [39] Fig. 1.

### Stage 1 of SEBA: systematic approach

A Population, Concept, and Context (PCC) approach was employed to guide the primary research question which is “*what is known about portfolios in postgraduate surgical education?*” and the secondary research questions are “*what are its components?*”, “*how are they implemented?*” and “*what are the strengths and weaknesses of current portfolios in postgraduate surgical education?*” The PICOS format was used to confine this review to ‘trainees’ which are doctors and residents undergoing surgical training (Table 1).

**Table 1:** PICOS, Inclusion Criteria and Exclusion Criteria Applied to Database Search.

**PICOS**	**Inclusion criteria**	**Exclusion criteria**
Population	• Junior doctors, residents, specialists and/or doctors and/or physicians in the surgical field • Postgraduate medical students undergoing surgical training	• Allied health specialities such as Pharmacy, Dietetics, Chiropractic, Midwifery, Podiatry, Speech Therapy, Occupational and Physiotherapy, Physician Assistants • Non-medical specialities such as Clinical and Translational Science, Alternative and Traditional Medicine, Veterinary, Dentistry
Intervention	• Papers that addressed the use of portfolios for junior doctors, residents, specialists and/or doctors and/or physicians and/or postgraduate medical students in the surgical field Criteria essential to be considered a portfolio: • A collection of evidence of learning across time • It must also include personal intellectual engagement with the content of the portfolio and associated learning • Interventions that meet the above criteria were included regardless of whether they referred to their interventions as portfolios All types of portfolios were considered in this study.	Other documentation methods or learning tools that are: • Not a collection but rather a singular piece of work • Only from a single time point • Does not include personal intellectual engagement with the content and associated learning (for instance, curriculum vitae, logbooks and the use of personal digital assistants)
Comparison	Papers that addressed the following comparisons were also included: • Comparison of the various uses of portfolios in different teaching settings • Comparison of the various types of portfolios • Comparison of teaching settings with and without portfolio-use • Evaluation of the effectiveness of portfolios in comparison to other educational interventions Papers that discussed portfolios without the above comparisons were also included.	NA
Outcome	Papers that measured the following outcomes were also included: • Impact of the use of portfolios on junior doctors, residents, specialists and/or doctors and/or physicians and/or medical students within the clinical, medical, research and/or academic settings • Impact of the use of portfolios on teaching • Impact of the use of portfolios on assessment • Impact of the use of portfolios on faculty	NA
Study design	• All study designs including: mixed methods research, meta-analyses, systematic reviews, randomized controlled trials, cohort studies, case-control studies, cross-sectional studies, descriptive papers, grey literature, opinions, letters, commentaries and editorials • Articles in English or translated to English • Year of Publication: 2000–2020	NA

Independent searches of were conducted on PubMed, SCOPUS, ERIC, Google Scholar, Embase, PsychInfo between 17th January 2021 and 14th February 2021, for articles published from 1st January 2000 to 31st December 2020. ‘Snowballing’ of references of the included articles ensured a more comprehensive review of the articles [40].

Using an abstract screening tool, the research team independently reviewed abstracts to be included and employed ‘negotiated consensual validation’ to achieve consensus on the final list of articles to be included [41].

### Stage 2 of SEBA: split approach

The split approach [42] sees concurrent thematic and directed content analysis of the included full-text articles by three independent teams. The first team summarised and tabulated the included full-text articles in keeping with recommendations drawn from Wong, Greenhalgh’s [43] RAMESES publication standards and Popay, Roberts’s [44] “Guidance on the conduct of narrative synthesis in systematic reviews” (Appendix A).

#### Thematic analysis

Using Braun and Clarke’s [45] approach to thematic analysis the second team analysed the included articles to find meaning and patterns in the data [46], [47], [48], [49], [50]. In phase two, ‘codes’ were constructed from the ‘surface’ meaning and collated into a code book to code and analyse the rest of the articles using an iterative step-by-step process [48]. As new codes emerged, these were associated with previous codes and concepts. In phase three, an inductive approach allowed themes to be “*defined from the raw data without any predetermined classification*” [49]. In phase four, the research team discussed their independent findings and employed “*negotiated consensual validation*” [41] to determine the final list of themes.

#### Directed content analysis

The third team employed Hsieh and Shannon’s [51] approach to directed content analysis in “*identifying and operationalizing a priori coding categories*” [51], [52], [53], [54], [55], [56] from “*Evaluating Competence Using a Portfolio: A Literature Review and Web-Based Application to the ACGME Competencies*” [57]. Any data not captured by these codes were assigned a new code [55]. ‘Negotiated consensual validation’ was used to achieve consensus on the final categories [54].

### Stage 3 of SEBA: jigsaw perspective

The Jigsaw Perspective employs Phases 4 to 6 of France et al. [58] adaptation of Noblit et al. [59] seven phases of meta- ethnographic approach to view the themes and categories as pieces of a jigsaw puzzle, where overlapping/complementary pieces are combined to create a bigger piece of the puzzle, referred to as themes/categories.

### Stage 4 of SEBA: funnelling

Themes/categories were compared with tabulated summaries [58,60], and included quality appraisals using MERSQI and COREQ [61,62]. The funnelled themes/categories created from this process forms the basis of the discussion's ‘line of argument’ in Stage 6 of SEBA.

## Results

**Fig. 2:**
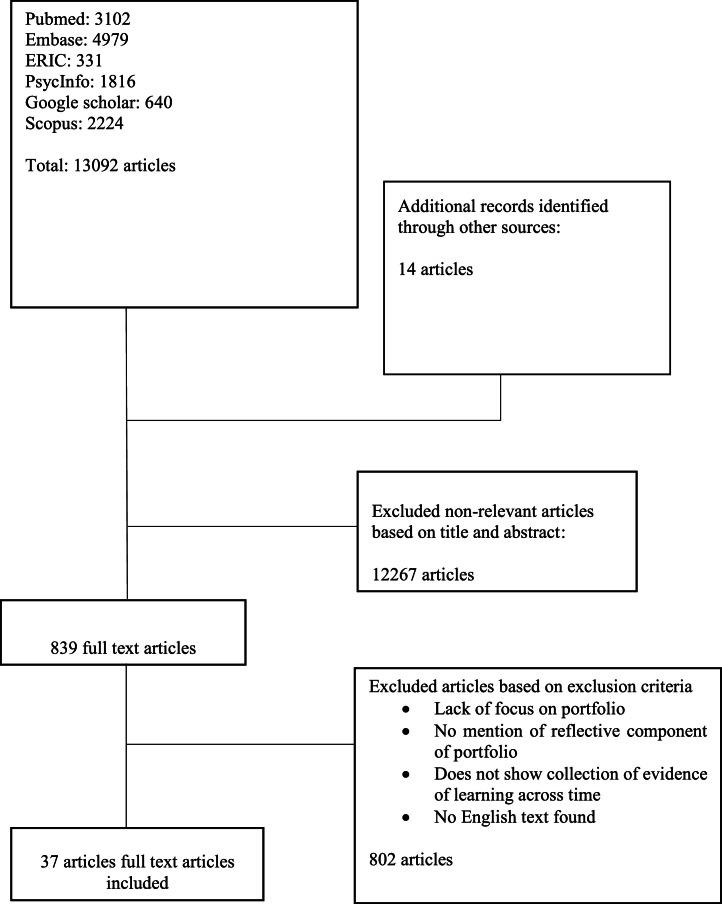
PRISMA Flowchart.

13,092 abstracts were reviewed, 839 full text articles were evaluated, and 37 articles were included (Fig. 2) where three funnelled domains were identified. These were the role of surgical portfolios, evaluation of surgical portfolios, and e-portfolios.

### Domain 1: role and strengths of surgical portfolios

Postgraduate surgical portfolios are focused on curating documentation of a trainee's competencies, achievements, experiences, feedback, and reflections. Secondary roles are summarised in Table 2.

**Table 2:** Roles and Strengths of Surgical Portfolios.

	**Subthemes**	**Elaboration/examples**
Roles	Documentation	• Trainee's progress over time (11, 63, 65, 70, 74, 77) • Learning experiences (11, 64, 65) ○ Case-based learning(11, 15, 64, 73, 93) ○ Critical incidents (12, 15) ■ Surgical complications ○ Personal learning projects within the context of PBLI (87) • Knowledge, attitudes and skills (15, 73) • Assessments and feedback (14, 65, [68], [69], [70], [71], [72]) ○ Mini-CEX(67, 69, 71, 72) ○ Case-based discussions(69, 71, 72) ○ Direct Observed Procedural skills(69, 71, 72) ○ Multi-source feedback(78) • Achievements (79, 82) • Official records such as certificates, transcripts and immunisation records (65, 66, 84)
	Evaluation of trainees	Through • Assessment of competencies (9, [13], [14], [15], [65], [66], [67]) ○ Practice-based learning and improvement (12, 15) ○ Work-place based assessments (69, 71, 72, 86) ○ Procedure-based assessments (PBA) (9, 72) ○ Non-technical skills for Surgeons (NOTSS) (9) ■ Situation awareness ■ Decision-making ■ Team working ■ Leadership
	Guided learning	• Set learning objectives (68, 82, 86, 89) • Map progress (14, 75, 82) • Recognising learning needs (89) • Ensure sufficient resources available (82) • Provide opportunities for development (17)
	Reflection	Portfolios enhance self-directed learning by stimulating reflection (11, 12, 15, 66, 73, 74, 80, 82, 87, 89, 93)
	Professional development	• Career development (66) • Continuous professional development (15, 64, 79, 93) ○ Life-long instrument for monitoring skills and competencies(63)
	Feedback	Sources of feedback Faculty (64, 73, 79) Supervisors (15, 71) Colleagues (78) Seniors (71)
		Role of feedback • Resident development (8) ○ Ensure progress towards desired outcomes (14, 75, 86) ○ Identifies specific strengths and weaknesses and hence training progression (14, 93, 94) • Promote further research into given topics (79)
		Feedback should beTimely (8) ○ Immediately after clinical encounter (8) ○ Regular intervals (63, 79, 94) ○ Weekly or biweekly (81)
		Types of feedbackNarrative (8, 78, 80) ○ More meaningful (8, 78, 80) Rating scales (14) • Bidirectional communication (13)
Strengths	Learner autonomy	Can be encouraged by • Ensuring active engagement in portfolio design (66) ○ Assigning responsibility to the learner to collect and maintain portfolio content (12, 63) • Having clear but flexible portfolio structure (66, 89) • Allowing flexibility (12, 15, 81) ○ In developing portfolio entries (73) ■ Based on relevance ■ Based on own's strengths and weaknesses ○ Setting learning outcomes (82) ○ To be expanded upon over time as residents gain experience (12) Supports learner ownership and avoids turning the portfolio into a bureaucratic exercise (66)
	Transparency	Established through • Clearly defined criteria for appraisal and assessment (63, 81, 82) ○ Identifying minimum competencies for training (81) Impact on learning • Reduce variations in quality of portfolios (71) • Guarantee core operative experience required (71)

### Domain 2: evaluation of surgical portfolios

A variety of methods are used to assess the reliability and validity, educational impact, acceptability and feasibility of surgical portfolios (Table 3).

**Table 3:** Evaluation of Surgical Portfolios.

**Subtheme**	**Elaboration/examples**
Validity and reliability	Practice-based assessments possess good validity and is a reliable method of assessing procedural skills (80)Reasons for poor validity and reliability in assessments • Evidence provided in portfolios are retrospectively assessed (9) • Record of experience does not necessarily equate with competence(9) ○ Submitted material does not correlate well with direct observation (9, 72) • Evaluation tends to be subjective (95) How to improve validity and reliability • Assessor training (17, 72, 79) • Achieve an optimal level of sampling spread across assessors, evaluation instruments and assessment contexts (13) ○ Use of quantitative and qualitative information from multiple sources (13) ○ Use as many different assessors as possible (72) • Provide descriptive assessment along with scores (80) • Standardisation of assessment process (17) ○ Use of clearer rubrics that are easier to understand, such as educationally-referenced scales (68)
Educational impact	By • Improving medical knowledge (79) ○ Demonstrate ACGME's practice-based learning and improvement and medical knowledge competencies (78) • Enhancing technical performance (8) • Providing learners with examples of improvement over time (12) Factors affecting educational value • Greater effort put into portfolios result in greater educational value (79) • Learning diminishes when regarded as a tick-box exercise (68)
Acceptability	Positive attitudes • High levels/improved levels of satisfaction (64, 79, 96) ○ Greater interests and higher satisfaction in newer residents compared to upper level residents (64) • Appreciation of value of portfolio (79) Negative attitudes • Viewed as a bureaucratic burden, resulting in demoralisation (14) • Poor quality of reflection ○ Inadequate (77) ○ Lacking in description (66) ○ Technical and cognitive instead of affective or interpersonal (71) • Concerns regarding ○ Relevance of learning objectives to trainee's professional role (17) ○ Validity and utility of educational tools contained by the portfolio (67) • General dissatisfaction due to ○ Lack of clear explanation of benefits (80) ○ Aspects of portfolio implementation (67)
Feasibility	• Time-consuming (73, [77], [78], [79], [80]) • Resource-intensive (81) ○ Requires direct observation by assessors (81) ○ Financial cost of upkeeping requiring long-term funding from ■ National funding (67) ■ Subsidies from deaneries (67) ■ Annual fees from participants (67) Impact on learning • Demotivation in usage (67) • Causes lack of meaningful reflection or educational value (64, 80) • Adversely impact training opportunities (86)

### Domain 3: E-portfolios

The use of e-portfolios are adopted for its accessibility, portability and convenience (Table 4).

**Table 4:** Modalities, Strengths and Limitations of Surgical E-portfolios.

**Subthemes**	**Elaboration/examples**
Modalities	Smartphone-based/ Tablet / Computer (8) Microsoft word-based (66) Email-based (79) Digital web-based (63, 65, 67)
Strengths of e-portfolios	Convenience (8, 14, 79, 82, 84, 87) • Lifts administrative burden (79) • Improves accessibility, enhancing motivation for trainee and trainer use (14, 79, 82, 87) • Allows for immediate evaluation by assessor (14) • Improves frequency of assessment by trainers, supplementing continuous appraisal of trainees’ performance (81) • Automation in updating data repository improves daily workflow (8, 79) Portability (14, 84)User-friendly (65, 79, 82)Tracks learners’ progress (14, 82, 84) • Improved organisation, aggregation and cataloguing of the multitude of residency performance artifacts (84) • Rapid access for trainees to report issues or progression (84) • Supervisors can keep track of trainee's progress instead of worrying about the short time trainees spend in OR (14) Comprehensiveness and inclusiveness (8, 65, 82) • Allows for a various types of documentation in one place ○ Eg. learning plans, evidence of progress, outcomes of assessments, online logbooks, and portfolios of other activities (82) • Can be applied to all clinical domains of resident performance (8) Interactive between trainer and trainee (65)
Limitations to e-portfolios	Technical burden (66, 73, 82) • Portfolio use is hampered by lack of familiarity with information technology (73) • Poor website navigability due to technical problems and poor construction (82) Bureaucratic burden (14) • Requires time from busy surgical trainers and trainees in order to adapt to the new way of learning • Lack of convincing validity ○ Difficult to quantify changes brought about by portfolio usage ○ Viewed as a tick-box exercise rather than an educational tool Issues with an open format (79) • Reduced sense of ownership to maintain one's portfolio • Reduced tendency to describe weaknesses (eg. mistakes, problems, embarrassing moments) Requires user training before using (81)

### Stage 5 of SEBA: analysis of evidence-based and non-data driven literature

The themes drawn from evidenced-based publications were compared with those from non-data based articles (grey literature, opinion, perspectives, editorial, letters), with the themes from both groups to be similar, and non-data based articles did not bias the analysis untowardly.

## Discussion

### Stage 6 of SEBA: synthesis of SSR in SEBA

In answering the primary research questions, this SSR in SEBA confirms portfolio's burgeoning reputation as a powerful tool to foster continuous personal and professional growth [63]; enhance self-directed learning [11,64,65]; capture the development of longitudinal surgical competencies [9,[13], [14], [15],[65], [66], [67]], curate the student's achievements (14, 65, [68], [69], [70], [71], [72]) and development of knowledge, skills, and attitudes [15,73]; collate written feedback [68,[72], [73], [74]]; and facilitate guided reflections [75]. However in answering its secondary research questions, this SSR in SEBA highlights variations in the quality, content and appropriateness of data included [76] and underscoring the varied roles played by portfolios [73,[77], [78], [79], [80], [81]].

By providing a platform for documenting, mapping and evaluating the trainee's longitudinal progress via multi-source feedback and assessments, surgical portfolios reiterate the notion that their professional development is a personalised experience. Whilst the surgical training and practice context, setting and culture will bring prerequisite structure to the portfolio, refinement must be led by mentors guiding the trainees as they co-design and scaffold the portfolios to best serve their learning needs.

These findings help demarcate the following wider considerations required of surgical portfolios, that they should be:

• longitudinal, potentially running from the beginning of medical school and throughout surgical training with opportunities for guided learning, reflection, professional development and streamlined feedback
• personalised and flexible, with learner autonomy encouraged to ensure active portfolio engagement and space for trainees to co-create their own unique professional identity within the confines of the stipulated requisite competencies
• assessment-driven, through the employ of multi-sourced micro-credentialling and validated assessments to inform further training, with remediation and refresher courses made available to ensure that learners achieve these necessary knowledge, skills and attitudes
• institution-led, with consistent oversight over learning progress to ensure timely and holistic mentoring support with clearly defined criteria for appraisal and evaluations to minimise variations in portfolio quality

While it is evident that the portfolio possesses many traits that render it ideal for the teaching and assessment, its ability to successfully fulfil these purposes is contingent upon proper implementation. With the growing adoption of e-portfolios in an increasingly digitally orientated society [82], the acceptability of portfolios are shifting away from time-consuming and resource intensive undertakings [16]. Lessons learnt from traditional forms of portfolios have already borne fruit. Beyond its flexibility in access, repository and content, e-portfolios offer increased user accessibility, better quality interactions and smoother facilitation, clarification, and feedback [81]. Simplified workflows and automatic updates have also reduced the administrative burden [73,[77], [78], [79], [80], [81]], whilst its portability has been seen to enhance clinical productivity, efficiency and quality [83] and program evaluations and oversight [8,84].

#### Implementation of a surgical E-Portfolio

With e-portfolios seen to be merely traditional portfolios applied on largely purpose built online platforms, the incorporation of portfolios into surgical training programs is contingent upon the willingness of host organizations to invest, support and implement these platforms [76,85]. Incumbent upon this are efforts to engage, train and support trainees and tutors on the use of these structures, particularly when the quality of portfolios are inherently tied to the quality of its entries, the extant of its content and the investment in time and effort by trainees [76,85]. The value of e-portfolios as a means of aiding reflection and feedback, enhancing cultural sensitivity and contextual considerations and inculcating institutional values and standards must be effectively communicated to users [76,85]. Indeed for e-portfolios to take up the mantle as a tool for learning, reflection and personal and professional growth, and influence the quality of patient outcomes, a structured approach that clearly defines the e-portfolio's role in a longitudinal training program is key [85].

The e-portfolio is, at its core, an assessment tool — an amalgamation of the various workplace based assessment tools that have been time-tested and validated for use in medical schools around the world [72]. Individually, through observation and interaction, these workplace based assessments evaluate the “day-to-day practices undertaken in the working environment” [86]. The synergy between mentorship and the multiple assessment tools allows for both learning and all-rounded assessment of an individual [82], which are then reviewed by either trained facilitators(72, 78), faculty members [64,79], council members [66], clinical professionals(63, 68, 72, 74, 82), peers or external reviewers [82,87], for making promotion-related decisions [84]. However, the ethos of portfolios, much like the workplace based assessments (WBA)s that they contain [8,9,72], should be seen to as a tool *for* development rather than assessments *of* growth and progress [85,86]. Thus whilst mini-clinical evaluation exercise, direct observation of procedural skills, discussion of clinical cases, and 360° assessments (also known as multi-source feedback), required by the Intercollegiate Surgical Curriculum Programme (ISCP) and four Surgical Royal Colleges (as methods to meet the trainee centred, service based, quality assured, flexible, coached, structured and streamlined training tenets adopted [67]), must be included [68], a mentored program will provide place and support for reflection and remediation, role modelling, coaching, supervision and career guidance [12,88,15]. Dedicated and trained mentors [63,75,80] would help programs meet Practice Based Learning and Improvement (PBLI) initiatives, guiding trainees as they “monitor, reflect and analyse practice experience; and identify, engage in, and apply improvements or new learning” [12,15]. Aside from ensuring a longitudinal perspective of training and development, mentoring programs would not only improve academic support, set and attain learning objectives, but boost confidence and enhance enjoyment and sense of belonging amongst trainees [68,82,86,89].

A further consideration for the employ of surgical e-portfolio must be effective IT training [90,91], support and expertise to support trainees and mentors [73,[77], [78], [79], [80], [81]]. Platforms must be accessible and robust and allow flexibility in the type of data included to ensure flexibility and personalization of the portfolio [72,73,81]. Trainees and mentors should also be provided with peer support to enhance portfolio effectiveness.

### LIMITATIONS

This review focused on information surrounding portfolios in postgraduate surgical education and excludes information that is not unique to the speciality. Hence, the information identified may not be generalisable or relevant to other specialities [92], and further studies are required to ascertain the evidence for portfolio use in each speciality.

Furthermore, only papers written in English were evaluated in this study. Hence, our findings may not be applicable in some countries with different socio-cultural contexts. As we have suggested in the use of portfolios using an electronic medium, there may be concerns in countries where such technologies are less readily available, hence, adaptations to our proposed portfolio implementation must be made.

## Conclusions

The data in this SSR in SEBA underscores the need for systematic assessment tools to evaluate e-portfolio as well as evaluate the programs as a whole. Evaluation of platforms and operating systems are required, as should careful guidance on context specific adaptations to the particular setting and different postgraduate surgical training requirements.

## Declarations

### Ethics approval and consent to participate

NA

### Consent for publication

NA

### Availability of data and materials

All data generated or analysed during this review are included in this published article [and its supplementary files].

### Authors’ contributions

All authors were involved in data curation, formal analysis, investigation, preparing the original draft of the manuscript as well as reviewing and editing the manuscript. All authors have read and approved the manuscript.

## Conflicts of Interest and Source of Funding

There are no competing interests. No funding was received.

## Appendix Supplementary materials

Image, application 1
