# Supplementary material for: Surgical portfolios: A systematic scoping review
Source: Surg Pract Sci. 2022 Jul 6;10:100107. doi: 10.1016/j.sipas.2022.100107 (PMC11749990; doi:10.1016/j.sipas.2022.100107)
Supplement: Supplementary file 1 [file mmc1.docx]

| Author/Year | Article title | Type of study | MERSQI | COREQ | Study Aim | Methodology | Key findings | Proposed solutions/conclusions |
| --- | --- | --- | --- | --- | --- | --- | --- | --- |
| Clay et al. 2007 | Development of a web-based, specialty specific portfolio | Descriptive | NA | NA | “This article illustrates the creation of a specialty specific portfolio that can be used by several different residency programs to document resident competence during a given rotation.” | “Three different disciplines (anesthesiology, surgery and medicine) worked together to create a critical care medicine portfolio. We began by reviewing the curriculum requirements for critical care medicine and organized these requirements into the six ACGME core competencies. We then developed learner led exercises in each core competency that were specific to critical care. Each exercise includes assessment of resident knowledge and application, an evaluation of the exercise, a learner self-assessment of skill, and a review of performance by a faculty member. Portfolio entries are highlighted in a multi-disciplinary weekly conference and posted on a critical care web site at our University.” | “The strengths of the specialty specific portfolio include the ability to be specific to the learning objectives of that rotation/specialty and uniform in content across multiple disciplines within that specialty. Commitment to the portfolio from multiple disciplines allows for careful refining of the portfolio and reduction of duplicative efforts. These specialty specific portfolios could potentially be improved upon if professional organizations were committed to their adaptation, perhaps as a component of maintenance of certification.  The portfolio entries as we have described are also useful for prompting frequent self reflection/evaluation and for providing trainees with feedback on their self-perceptions. Self assessment, a skill which is not often taught or evaluated in medical education, is notoriously inaccurate. However, self assessment may be improved with deliberate practice, and frequent self assessment with feedback on that self assessment.  If other specialties were to create similar portfolios, the logistics of creating a portfolio for each resident would be simplified: the portfolio could be built a rotation at a time, meeting the objectives of that rotation monthly, and meeting the ACGME requirements over the course of a residency. Furthermore, by simply requiring a certain number entries from each core competency by the end of each year or the end of a residency, the learner is given the discretion to choose those exercise which he/she feels is most relevant given the cases he/she has encountered.” | “Creation of specialty specific portfolio reduces redundancy between disciplines, allows for increased time to be spent on the development of exercises specific to rotation objectives, and aids program directors in the collection of portfolio entries for each resident over the course of a residency.” |
| Dekker et al. 2009 | Mentoring portfolio use in undergraduate and postgraduate medical education | Qualitative/quantitative | 8 | 16 | “Mentoring is widely acknowledged as being crucial for portfolio learning. The aim of this study is to examine how mentoring portfolio use has been implemented in undergraduate and postgraduate settings.” | “The results of interviews with six key persons involved in setting up portfolio use in medical education programmes were used to develop a questionnaire, which was administered to 30 coordinators of undergraduate and postgraduate portfolio programmes in the Netherlands and Flanders.” | “The interviews yielded four main aspects of the portfolio mentoring process – educational aims, individual meetings, small group sessions and mentor characteristics. Based on the questionnaire data, 16 undergraduate and 14 postgraduate programmes were described. Providing feedback and stimulating reflection were the main objectives of the mentoring process. Individual meetings were the favourite method for mentoring (26 programmes). Small group sessions to support the use of portfolios were held in 16 programmes, mostly in the undergraduate setting. In general, portfolio mentors were clinically qualified academic staff trained for their mentoring tasks.” | “​​The mentoring process within portfolio programmes has a strong focus on providing feedback and stimulating reflection. Students are mainly mentored individually. In addition, some programmes provide small group sessions to offer students an opportunity to discuss experiences and practise reflective skills. Some institutions also facilitate peer meetings for their portfolio mentors to share experiences and expertise. This study reveals that Dutch and Flemish medical education programmes show a wide variety in why, what, where and how the mentoring process in relation to portfolio use is carried out. Further research should elucidate the most effective way of mentoring portfolio use.” |
| Overeem et al. 2010 | Three methods of multi-source feedback compared: A plea for narrative comments and coworkers’ perspectives | Quantitative | 10 | NA | “To compare three methods of MSF for consultants in the Netherlands and evaluate the feasibility, topics addressed and perceived impact upon clinical practice.” | “In 2007, 38 facilitators and 109 consultants participated in the study. The performance assessment system was composed of (i) one of the three MSF methods, namely, Violato's Physician Achievement Review (PAR), the method developed by Ramsey et al. for the American Board of Internal Medicine (ABIM), or the Dutch Appraisal and Assessment Instrument (AAI), (ii) portfolio, (iii) assessment interview with a facilitator and (iv) personal development plan. The evaluation consisted of a postal survey for facilitators and consultants. Generalized estimating equations were used to assess the association between MSF method used and perceived impact.” | “It takes on average 8 hours to conduct one assessment. The CanMEDS roles ‘collaborator’, ‘communicator’ and ‘manager’ were discussed in, respectively, 79, 74 and 71% of the assessment interviews. The ‘health advocate role’ was the subject of conversation in 35% of the interviews. Consultants are more satisfied with feedback that contains narrative comments. The perceived impact of MSF that includes coworkers’ perspectives significantly exceeds the perceived impact of methods not including this perspective.” | “Performance assessments based on MSF combined with a portfolio and a facilitator-led interview seem to be feasible in hospital settings. The perceived impact of MSF increases when it contains coworkers’ perspectives.” |
| Green et al. 2017 | Role of video documentation and video portfolios for surgical trainees | Quantitative | 7 | NA | “This study investigated the perception of usefulness of video for documenting basic surgical skill competency in general surgery and surgical specialties.” | “Program and assistant program directors at our institution in ophthalmology, orthopaedics, otolaryngology, urology, oral and maxillofacial, general, and plastic surgery received an anonymous 8-item survey, including an example video clip of an intern completing a vertical mattress suture. Respondents answered questions about how various basic skills pertained to their specific specialties, potential benefits of video documentation as demonstrated in the example clip, and current practices for documenting skill competency.” | “Response rate was 88% (14/16), and all felt skills highlighted in the video, such as needle handling and knot tying, were “somewhat” or “very” relevant to their subspecialty. Respondents agreed (64% to 79%) that competency in 2-handed tie, 1-handed tie, tie without tension, subcuticular suture, horizontal suture, and vertical suture was relevant to their specialty. Sixty-four percent valued video documentation of interns’ skills. They endorsed these potential uses of videos: ongoing documentation (78%), tailoring residents’ operative experience (64%), and developing remediation plans (64%). No documentation of resident skill prior to operating room exposure was reported by 79%. Written comments were positive about video documentation.” | “This study suggests that surgical knot tying and suturing skills are relevant, and portfolios with videos of these skills may be useful to demonstrate progression toward competency. Future employers may request these portfolios to evaluate surgeons’ qualifications.” |
| Green 2016 | An Innovative, No-cost, Evidence-Based Smartphone Platform for Resident Evaluation | Quantitative | 11 | NA | “Timely performance evaluation and feedback are critical to resident development. However, formulating and delivering this information disrupts physician workflow, leading to low participation. This study was designed to determine if a locally developed smartphone platform would integrate regular evaluation into daily processes and thus increase faculty participation in timely resident evaluation.” | “Formal, documented resident operative and patient interaction evaluations were compiled over an 8-month study period. The study was divided into two 4-month phases. No changes to the existing evaluation methods were made during Phase 1. Phase 2 began after a washout period of 2 weeks and coincided with the launch of a smartphone-based platform. The platform uses a combination of Likert scale questions and the Dreyfus model of skill acquisition to describe competence levels in technical and nontechnical skills. The instrument inflicts minimal effect on surgeon workflow, with the aim of integrating resident evaluation into daily processes. The number of different faculty members performing evaluations, resident level (postgraduate year), type of interaction or procedure, and competency data were compiled. All evaluations were tracked by the program director as they were automatically uploaded into a database. Faculty members were introduced to the new platform at the beginning of Phase 2, and previous methods of evaluation continued to be encouraged and were considered valid throughout both phases of the study. Data were analyzed using Fisher exact test for specific PGY level, and chi-square test was used for overall program analysis. Statistical significance was set at p < 0.05.” | “Total faculty engagement, that is, number of faculty members completing evaluations, increased from 13% (5/38) in Phase 1 to 53% (20/38) in Phase 2. During Phase 1, all evaluations consisted of online forms through the department’s established system or e-mails to the program director. Evaluations were completed in 0.9% (15/1599) of cases residents completed in Phase 1 versus 12% (217/1812) of those in Phase 2. During Phase 2, evaluations were conducted exclusively using the new platform. This was done based on participant’s choice. Total numbers of residents and core faculty members did not change between Phases 1 and 2.” | “A smartphone-based platform can be created with existing technology at no cost. It is adaptable and can be updated in real-time and can employ validated scales to build an evaluation portfolio for learners assessing technical and nontechnical skills. Furthermore, and perhaps most importantly, it can be designed to integrate into existing workflow patterns to increase faculty participation.” |
| Hassan 2011 | Use of Structured Portfolio in Surgical Training of Postgraduate Medical Education | Qualitative/quantitative | 7.5 | 8 | This study was designed to determine if a locally developed smartphone platform would integrate regular evaluation into daily processes and thus increase faculty participation in timely resident evaluation. | Formal, documented resident operative and patient interaction evaluations were compiled over an 8-month study period. The study was divided into two 4-month phases. No changes to the existing evaluation methods were made during Phase 1. Phase 2 began after a washout period of 2 weeks and coincided with the launch of a smartphone-based platform. The platform uses a combination of Likert scale questions and the Dreyfus model of skill acquisition to describe competence levels in technical and nontechnical skills. The instrument inflicts minimal effect on surgeon workflow, with the aim of integrating resident evaluation into daily processes. The number of different faculty members performing evaluations, resident level (postgraduate year), type of interaction or procedure, and competency data were compiled. All evaluations were tracked by the program director as they were automatically uploaded into a database. Faculty members were introduced to the new platform at the beginning of Phase 2, and previous methods of evaluation continued to be encouraged and were considered valid throughout both phases of the study. Data were analyzed using Fisher exact test for specific PGY level, and chi-square test was used for overall program analysis. Statistical significance was set at p < 0.05. | Total faculty engagement, that is, number of faculty members completing evaluations, increased from 13% (5/38) in Phase 1 to 53% (20/38) in Phase 2. During Phase 1, all evaluations consisted of online forms through the department’s established system or e-mails to the program director. Evaluations were completed in 0.9% (15/1599) of cases residents completed in Phase 1 versus 12% (217/1812) of those in Phase 2. During Phase 2, evaluations were conducted exclusively using the new platform. This was done based on participant’s choice. Total numbers of residents and core faculty members did not change between Phases 1 and 2. | A smartphone-based platform can be created with existing technology at no cost. It is adaptable and can be updated in real-time and can employ validated scales to build an evaluation portfolio for learners assessing technical and nontechnical skills. Furthermore, and perhaps most importantly, it can be designed to integrate into existing workflow patterns to increase faculty participation. |
| Karthikeyan & Pulimoottil 2018 | Design and Implementation of Competency Based Postgraduate Medical Education in Otorhinolaryngology: The Pilot Experience in India | Descriptive | NA | NA | “This paper details the first concrete attempt at establishing a CBME curriculum in Otorhinolaryngology in India.” | “The design and implementation of the CBME curriculum was carried out in four phases, in a time-bound manner over a period of 6 months. Phase I consisted of an extensive literature review and a clarification of the major objectives of the program. Phase II involved the listing out of 20–30 entrustable professional activities (EPAs) for each specialty and the 13 core EPAs common to all incoming residents and the subsequent mapping of these EPAs to their respective domains of competence and year-wise levels of competence. This was followed by the development of milestones for each EPA and appropriate clinical vignettes. Phase III focused on development of 360° assessment strategies, including the in-house development of an e-portfolio. Phase IV was dedicated to the implementation of the CBME curriculum, and involved various sensitization and orientation programs for faculty and the new residents.” | “This exercise in designing and implementing a CBME program showed the important role that intra-departmental and inter-institutional cross-communication and exchange of ideas vies-a-vie workshops and personal communication play in bridging the lapses in knowledge in this emerging area, reaching consensus to achieve project goals and for finding relevant solutions to common problems. “ | “Medical education in India presents its own peculiar set of logistical and cultural challenges. Keeping in line with the recommendations of the Medical Council of India regarding Postgraduate Medical Education, it is essential that medical colleges in India not fall behind the international paradigm shift towards CBME.” |
| Khan 2008 | Evaluation of the educational environment of postgraduate surgical teaching | Quantitative | 10.5 | NA | “The study was conducted to determine the change if any in the education environment of postgraduate surgical teaching in a leading teaching hospital in London when a teacher-centred, old-fashioned postgraduate teaching approach was replaced with a student-centred, self-assessment, portfolio-based approach” | “Postgraduate Hospital  Educational Environment Measure (PHEEM): Twenty postgraduate trainees filled in the questionnaire before and after the change in their learning/ teaching pattern” | “The response rate was 100%.  No statistically significant difference in the overall score for the two teaching environments (p=0.8024,  95% CI= -5.549273 to 4.349273) was found, because the loss of on-call rooms, trainee’s mess and  catering services statistically significantly deteriorated the social support subscale of the PHEEM scale  (p<0.0001, 95% CI=6.66752 to 13.03248) to counteract any statistically significant improvement in the  teaching role perception subscale of the instrument (p=0.001, 95% CI= -12.443896 to -4.856104).  There was no statistically significant difference in the role autonomy perception subscale in the two  methods (p=0.3663, 95% CI= -5.870437 to 2.270437).” | “A student-centred approach to postgraduate teaching is better than a teacher-centred approach. However, further studies will be needed to evaluate both postgraduate teaching and training environment.” |
| Schmitz et al. 2010 | Establishing a usable electronic portfolio for surgical residents: trying to keep it simple | Descriptive | NA | NA | “Elaborate web-based portfolios may not be needed for resident  evaluation and career development. An approach for busy academic medical departments is described. “ | NA | “We feel that the portfolio needs to be and should be tailored to the needs of the program and that of its residents. The inherent simplicity of this approach is that it is incredibly flexible and has the ability to be tailored to the needs of the end user, just as one would modify a document or a presentation.” | Keeping portfolios technologically simple has resulted in a “win-win” situation for the department and our residents. Active resident engagement in portfolio design led to a better product that  residents feel reflects their individuality and benefits their careers yet serves departmental needs for systematic assessment and documentation of ACGME core competencies. |
| Takayesu et al. 2012 | Assessing patient care: summary of the breakout group on assessment of observable learner performance | Qualitative | NA | 7 | In this article, the authors review each method's supporting reliability and validity evidence and make specific recommendations for future educational research. | “A search was conducted using MEDLINE 1996-present using the key word search terms “assessment,” “patient care,” “competency,” “competence,” “assess*,” “emergency,” and “education” and limited to humans and English language [boolean search: ((assessment and patient care AND (competency or competence)) OR (assess* and emergency and education) resulting in 3493 hits; (patient care and competency) and assessment resulting in 282 references]. These searches were combined with the additional search terms “resident* or medical student*” (58,880) resulting in 414 and 267 final results, respectively. After reviewing for relevance, 76 articles remained. Additional references were identified from review of these results and are included when relevant. These articles were used as a foundation for the breakout group's discussion.” | “Direct observation, OSCE, and HFS have the strongest evidence as valid and reliable assessment methods. Global assessments and 360-degree evaluations require specific behavioral anchors to increase their validity and large response rates to control for confounders such as the halo/millstone effects and individual rater variability. Metrics can provide valuable performance data for residents in their more senior years, since these measures can be directly compared to attending physician performance standards. Portfolios and self-reflection lack evidence to support their use as stand-alone assessments of patient care, but have the benefit of encouraging the reflective and learner-directed practice that forms the basis of continuing medical education.” | “A holistic assessment of competence in patient care requires a mixture of methods rather than any single method of assessment, taking into account each method's costs, benefits, and current level of evidence. Assessments should focus on specific behaviors, tasks, and skills, with opportunities for formative feedback and repeated performance, enabling formative feedback to drive learner growth. The assessment rubric should undergo rigorous testing of its reliability and evidence of validity by comparing its results to actual patient care and patient outcomes.  Follow-up assessment is important to ensure durability of competence, which can influence curricular changes in the timing, structure, or repetition of educational interventions throughout residency training. A variety of assessment methods is necessary to accommodate local variations in access to high-cost technologies such as HFS.” |
| Webb et al. 2006 | The Surgical Learning and Instructional Portfolio (SLIP) as a self-assessment educational tool demonstrating practice-based learning | Quantitative | 8.5 | NA | “The authors describe the evolution of the Surgical Learning and Instructional Portfolio (SLIP) into a worthwhile educational tool.” | “In March 2001, the authors began a program to encourage residents to develop a case-based portfolio to document their experience and demonstrate acquisition of knowledge in caring for a variety of surgical diseases. The monthly case topic was chosen by the resident and reported using a template: case history, supporting diagnostic studies, differential diagnosis, final diagnosis with ICD-9 coding, management options, treatment used, 3 lessons learned, embellishment of 1 lesson, and 2 articles supporting the experience. Initially, cases were submitted to the program coordinator and reviewed every 6 months with a faculty advisor to provide feedback.” | “After the first 18 months of this program, resident compliance was less than 50%, satisfaction was low, and formal review did not occur. In July 2004, a single faculty member became responsible for evaluating and providing feedback on the monthly SLIPs. The assignments were handled electronically with feedback delivered within the month via e-mail. SLIP quality as measured by resident compliance and satisfaction improved.” | “These SLIPs have matured into a valuable educational tool satisfying multiple ACGME competencies. This portfolio system required direct faculty feedback to become successful.” |
| Webb & Merkley 2012 | An evaluation of the success of a surgical resident learning portfolio | Qualitative | NA | 14 | “This study was performed to evaluate the SLIP program using resident and faculty perspectives in the domains of satisfaction, compliance, and educational value.” | “Likert scale surveys were distributed to residents to assess satisfaction. Using a semistructured format with subsequent qualitative analysis of the meeting transcript, a focus group discussion was held with the SLIP director, SLIP facilitator, and program coordinator. An analysis of the program compliance was performed by review of SLIP entry dates. Finally, the quality of the SLIP entries (n = 420) was analyzed in a blinded manner using a locally developed standardized SLIP assessment tool. Data analysis was performed using Pearson's correlation and Cronbach's alpha.” | “Residents were satisfied with the program and felt the Web-based format promoted self-reflection. They perceived that time spent was appropriate. Residents also believed they gained medical knowledge of their own specific entry topics but did not learn routinely from others' entries. Faculty asserted that the Web-based platform eased the administrative burden but did not necessarily alter the quality of the SLIP entries. Compliance with the assignment was 100%. SLIP entry analysis demonstrated the reflection and understanding of the topics chosen. However, the overall quality assessment of entries was hindered by suboptimal interrater reliability (inter-rater reliability (IR) = 0.636).” | “The SLIP program allows residents to demonstrate practice-based learning and improvement of medical knowledge. The Web-based format provides transparency and ease of administration. Quality assessment of individual portfolio entries remains a challenge to the widespread adoption of portfolios.” |
| Webb et al. 2014 | Assessing competency in practice-based learning: a foundation for milestones in learning portfolio entries | Qualitative | NA | 15 | “The purpose of this study is to identify criteria used by surgical educators to judge competence in Practice-Based Learning and Improvement (PBL&I) as demonstrated in learning portfolios.” | “A total of 6 surgical learning and instructional portfolio entries served as documents to be assessed by 3 senior surgical educators. These faculty members were asked to rate and then identify criteria used to assess PBL&I competency. Individual interviews and group discussions were conducted, recorded, and transcribed to serve as the study dataset. Analysis was performed using qualitative methodology to identify themes for the purpose of defining competence in PBL&I. The assessment themes derived are presented with narrative examples to describe the progression of competency.” | “The collaborative coding process resulted in identification of 7 themes associated with competency in PBL&I related to surgical learning and instructional portfolio entries: (1) self-awareness regarding effect of actions; (2) identification and thorough description of learning goals; (3) cases used as catalyst for reflection; (4) reconceptualization with appropriate use and critique of cited literature; (5) communication skills/completeness of entry template; (6) description of future behavioral change; and (7) engagement in process—identifies as personally relevant.” | “The identified themes are consistent with and complement other criteria emerging from reflective practice literature and experiential learning theory. This study provides a foundation for further development of a tool for assessing learner portfolios consistent with the Accreditation Council for Graduate Medical Education’s Next Accreditation System requirements.” |
| Phillips & Madhavan 2013 | A Critical Evaluation of the Intercollegiate Surgical Curriculum and Comparison With its Predecessor the ‘‘Calman’’ Curriculum | Descriptive | NA | NA | “The aim of this review is to examine the changes to the curriculum and effect on surgical training.” | “A comparison was made of the Calman Curriculum and the ISCP and how they met training needs.” | “The new curriculum is multifaceted, providing a more prescriptive detail on what trainees should achieve and when, as well as allowing portfolio, learning agreements, and work-based assessments to be maintained on an easily accessed website. The increasing emphasis on work-based assessments has been one of the major components, with an aim of providing evidence of competence. However, there is dissatisfaction amongst trainees with this component which lacks convincing validity.” | “This new curriculum significantly differs from its predecessor which was essentially just a syllabus. It needs to continuously evolve to meet the needs of trainees whose training environment is ever changing.” |
| Pereira & Dean 2013 | British Surgeons’ Experiences of a Mandatory Online Workplace Based Assessment Portfolio Resurveyed Three Years On | Quantitative | 8 | NA | An online portfolio, the Intercollegiate Surgical Curriculum Programme, became mandatory for British surgical trainees 5 years ago, alongside a compulsory £125 (US$200) annual fee. It was previously demonstrated that there was widespread dissatisfaction with its 2008 ver. 5. The article seeks to evaluate and contrast user satisfaction with ver. 8. | “A total of 359 users across all surgical specialties and UK regions were randomly sampled and surveyed in 2011 by online questionnaire regarding ISCP, elogbook (http://www.elogbook.org.uk), and results compared with 539 users surveyed in 2008. Likert 5-point rating scales were largely used and data analyzed using χ2 tests.” | “Seventy-nine percent used ISCP and 38% elogbook (http://www.elogbook.org); 201 responders (56%) evaluated ISCP ver. 8; 31% had registered for at least 1 year, and 59% for 3 years or more. Modal ratings were “average” throughout, with the following percentages of responders rating “poor” or worse vs “good” or better the following domains: registration 12% vs 35%; induction procedure 41% vs. 15%; workplace based assessments 36% vs 22%; peer assessment tool 34% vs 25%; recording meetings 34% vs 19%; Helpdesk 11% vs 40%; user friendliness 29% vs 24%. Trainees were neutral about ISCP's impact upon training and 44% thought that ISCP was needed. Statistically significant (p < 0.05) improvements were seen in user satisfaction with ISCP throughout all domains comparing ver. 8 (2011) to ver. 5 (2008).” | “The performance of ISCP has improved in the 4 years since its inception with proportionately less negative feedback. British surgeons remain dissatisfied with several of its tools, in particular its workplace-based assessments. Half a decade on, these assessments remain without appropriate evidence of validity despite increasing demands upon trainees to complete quotas of them. With reduced permitted training hours, the growing online bureaucratic burden continues to demoralize busy surgical trainers and trainees.” |
| Alkhayal et al. 2012 | Toward an objective assessment of technical skills: a national survey of surgical program directors in Saudi Arabia | Qualitative/quantitative | 13.5 | 8 | “We conducted this study to document the assessment tools for technical skills currently used in different surgical specialties, their relationship with remediation, the recommended tools from the program directors' perspective, and program directors' attitudes toward the available objective tools to assess technical skills.” | “This study was a cross-sectional survey of surgical program directors (PDs). The survey was initially developed using a focus group and was then sent to 116 PDs. The survey contains demographic information about the program, the objective assessment tools used, and the reason for not using assessment tools. The last section discusses the recommended tools to be used from the PDs' perspective and the PDs' attitude and motivation to apply these tools in each program. The associations between the responses to the assessment questions and remediation were statistically evaluated.” | “Seventy-one (61%) participants responded. Of the respondents, 59% mentioned using only nonstandardized, subjective, direct observation for technical skills assessment. Sixty percent use only summative evaluation, whereas 15% perform only formative evaluations of their residents, and the remaining 22% conduct both summative and formative evaluations of their residents' technical skills. Operative portfolios are kept by 53% of programs. The percentage of programs with mechanisms for remediation is 29% (19 of 65).” | “The survey showed that surgical training programs use different tools to assess surgical skills competency. Having a clear remediation mechanism was highly associated with reporting remediation, which reflects the capability to detect struggling residents. Surgical training leadership should invest more in standardizing the assessment of surgical skills.” |
| Borel-Rinkes et al. 2008 | Surgical training in the Netherlands | Descriptive | NA | NA | The article describes the modernisation of surgical training in the Netherlands. | NA | “Prerequisite for the successful implementation of this new program is the mandatory use of a uniform, digital, web-based (as opposed to hospital-based) portfolio. This is owned and managed by the resident and includes all aspects of the training program, including operations performed, tests taken, courses and conferences followed, and assessment. The portfolio will be a life-long instrument for monitoring the skills and competence of the surgeon. Assessment forms should be uniform, standard, and easy to fill out digitally by the member of the surgical team conducting the assessment. Feedback may thus be given at regular intervals by the program director based on multiple assessments by various members of the surgical training team, thereby minimizing the risk of subjectivity. Hence, an essential part in the implementation of the plan is the professionalization of both surgeons/program directors and residents regarding their role in personal coaching and monitoring of the resident’s progress. Training sessions are currently being set up throughout the country for this purpose. These may also help ensure broad support for this ambitious modernization of surgical training. This new approach to surgical training will ask for an enormous effort on the part of surgeons and residents. The implementation of the new training tools and assessment in daily practice will be challenging but will result in more clearly defined surgical portfolios.” | “Within the next 6–12 months, the Dutch training in surgery will be restyled as outlined. This will create an even safer and more open training environment of high quality, with emphasis on a competency-driven program, regular feedback using standard tools, stored in a uniform, resident-owned digital portfolio. It will formalize the role of all members of the surgical team, as well as that of the program director and of the resident him or herself. Also, it will further underline the position of the surgeon as a team player. The government has implemented regulation for financing surgical training. However, not all financial issues of the medical specialty training program are resolved. The increasing influence of the government on financial aspects of medical specialty training will undoubtedly have profound effects, among others on the control of influx of new residents. The content of training is a definite matter of the professionals, and the role of the Association of Surgeons of the Netherlands and its training committee (the Concilium, Utrecht, The Netherlands) in describing and conducting both the training and its quality control should be guarded with caution.” |
| Brigley & Jasper 2010 | Evaluation of a multidisciplinary faculty to support learning in surgical practice | Qualitative | NA | 4 | Evaluate the Theatre Faculty Project | “The purpose of the evaluation was formative and summative: to produce judgements of worth of TFP principles and approaches and to present evidence that would inform decisions of hospital management on the development of TFP. It was therefore important that the inquiry was conceived, implemented and reported by independent researchers with varied experience in education and healthcare.  In evaluating the complex layers of the programme, a multi-method inquiry was adopted, that integrated the perspectives of key stakeholders. It comprised: structured observation of six "live" seminars, DVD recordings of two seminars and a day-case TA; a focus group with six theatre staff; and one-to-one interviews with two surgeons and two trainees. The interviews and observation were conducted by the project director; the focus group was moderated by both evaluators. The evaluators jointly reviewed the TFP portfolios (including CRW) of four theatre staff, and CRW extracts of two other theatre staff, three surgeons and three trainees.  Interview transcripts were analyzed by the project director. Analysis of the portfolios and CRW, and of the focus group transcript, was conducted separately then triangulated by the evaluators. The data were subjected to a matrix analysis, with each matrix representing a progressive iteration in terms of the content and originator of data. The sequence involved: an initial listing of constructs; determination of their significance; identification of clusters or inter-related constructs; statements of emergent patterns or themes. Emergent themes in each data set were cross-referenced by the evaluators. Data collection was internally audited in ongoing team meetings and interim reports.  In this discussion of the evaluation, participants are identified by group and number (e.g., Surgeon 1). The focus is on interprofessional learning in the TFP seminars, rather than the implementation of TAs. In exploring both participants' perceptions and their multi-layered learning, the discussion moves from Level 1 (reactions) to Level 2 (learning outcomes) of educational evaluation” | “The evaluation found that TFP was exceptional in bringing surgeons, theatre staff and trainees together in CPD activities. The theatre staff, in particular, felt enthused and occasionally empowered by the inclusivity of seminar and practice activities. The putative benefits of establishing a theatre faculty were recognized, as was the place of the faculty concept in wider regulatory and quality frameworks of specialty training. Participants were now more aware of the complexity and value of their professional knowledge and practice, increasing their sense of professional worth and motivation to engage in dialogue with learning surgeons. In general terms, they understood the nature and importance of CRW and the invisibles, and how these might be developed with surgical trainees. Portfolio building, though time consuming, could be enjoyable and illuminative of the owner's educational and professional practice. For surgical trainees, the portfolios furnished an essential record of learning and progression in surgical training.” | “At the time of writing, the future of TFP was unclear. The multiple perspectives of this evaluation show that the aims, approaches and outcomes of TFP conveyed much that was of common professional value and of relevance to the local and national context of surgical education. With regard to the formative value of TFP, there is an urgent need to build on the programme's achievements, taking account of internal refinements that will engage more fully the multidisciplinary understandings and collective professional experiences of participants. In summative terms, it appears that the advances achieved thus far will count for little unless the host institution lends its weight to this important development and cements the faculty into its organizational structures.” |
| Rao et al. 2012 | e-Portfolio Competency Metadata: Pilot Study for a Call to Action | Qualitative | NA | 4 | “The six competency domains required by the Accreditation Council for Graduate Medical Education (ACGME) have led to a proliferation of measurement tools, assessment methods, and all forms of data from paper to electronic. The need exists to develop a standardized electronic (e)-portfolio to provide the aggregate data to improve education and patient care. This process requires a sound methodology using XML metadata to allow portability of e-portfolio data. “ | Publicly available metadata were surveyed and an e-portfolio system for the Henry Ford Hospital General Surgery Residency Program was developed. | “The information technology revolution has heralded a new era in health care. The transition from paper to electronic data-driven organizations in medical education as well as patient care brings about both challenges and opportunities While the ACGME developed the six core competencies to ensure that residency education adapts to shifting health care paradigms and provides goal-oriented standards to measure a training program’s effectiveness, we now need assimilation of electronic tools as part of the delivery and measurement, as well as portability, of these core competencies.  Our pilot of an e-portfolio system for the Henry Ford Hospital General Surgery Residency Program demonstrates that metadata tags allow for aggregate assessment information to be determined for the individual resident, program, and institution. The use of aggregate assessment data is necessary to drive educational change within programs and institutions, and is also important in residents’ self-reflection regarding their progress and goals. Similar metadata tags can be adopted in an e-portfolio for maintenance of certification and professional competence standards.  The implementation of our e-portfolio system allows rapid access to a variety of resident data categories and files for residency directors during annual site visits by ACGME reviewers. In the future, we will further refine reporting based on ACGME competencies.” | “While resident portfolios are often used to both assess and aim teaching toward the ACGME competencies, studies have highlighted the wide variability in portfolio quality and utilization. We believe the ACGME, in collaboration with its Residency Review Committees and in partnership with the ABMS, should establish a method to formalize and develop a standard for residency competency metadata. The metadata schema will provide a foundation for the development of e-portfolios that are portable and follow best practice implementation and assessment guidelines. By allowing the collection and reporting of aggregate data, this approach will allow us to become data-driven organizations and improve medical education and, ultimately, patient care outcomes.” |
| McCarthy & Kelly 2008 | Preparing a surgeon’s portfolio | Descriptive | NA | NA | This article reviews what in particular surgical trainees should place in their folders, the importance of the Intercollegiate Surgical Curriculum Project, and advice on the dos and don’ts of how to set up a folder/portfolio. | NA | NA | The surgeon’s portfolio is now an essential requirement for all trainees and trainers. It should demonstrate the trainee’s progression throughout training by highlighting satisfactory completion of the relevant competencies and the trainee’s ability to reflect on his/her experiences. The surgeon’s portfolio is not a static concept and is an ever-evolving dynamic medium that should be updated on an almost daily basis. In the future there will be greater emphasis on web-based portfolios. |
| Peeraer 2015 | The Development of an Electronic Portfolio for Postgraduate Surgical Training in Flanders | Descriptive | NA | NA | This article evaluates the development of electronic portfolio. | “The four Flemish surgical coordinators, together with experts from different universities, devised an  electronic portfolio. This portfolio holds both the logbook, as imposed by the evaluation committee and assessment  instruments used for the Master in Specialized Medicine. “ | “The e-portfolio is now used by a number of surgical trainees and has been approved by the evaluation committee. In 2015, all Flemish surgical trainees will be using one and the same e-portfolio.” | “Although the e-portfolio for surgical training has now been devised and accepted by all major parties  involved, a lot of work has to be done to implement the instrument. As resident duty hours show no improvement on  education in surgery (but rather a perception of worsened education) surgery training is fazing huge challenges.” |
| Webb & Merkley 2011 | The surgical learning and instructional portfolio: what residents at a single institution are learning | Qualitative | NA | 15 | A thematic content analysis of 50 random portfolio entries was conducted to identify lessons learned. | “Each month, all residents in our surgery residency program submit entries into their individual Surgical Learning and Instructional Portfolio (SLIP). The SLIP entries from July 2008 to 2009 (n = 420) were deidentified and randomized using a random number generator. We conducted a thematic content analysis of 50 random portfolio entries to identify lessons learned. Two independent raters analyzed the “3 lessons learned” portion of the portfolio entries and identified themes and subthemes using the constant comparative method used in grounded theory.” | “The collaborative coding process resulted in theme saturation after the identification of 7 themes and their subthemes. Themes in decreasing order of frequency included complications, disease epidemiology, disease presentation, surgical management of disease, medical management of disease, operative techniques, and pathophysiology. Junior residents chose to focus on a broad array of foundational topics including disease presentation, epidemiology, and overall management of diseases, whereas postgraduate year-4 (PGY-4) and PGY-5 residents most frequently chose to focus on complications as learning points.” | “Lessons learned reflect perceived needs of the trainees based on training year. When given a template to follow, junior and senior residents choose to reflect on different subject matter to meet their learning goals.” |
| Abdelaal 2020 | Procedure-based assessments: The past, the present and the future | Descriptive | NA | NA | In this review, the author aims to shed some light on my perspective on PBA, its values, limitations and concerns that have risen as a result of its introduction. The author also aims to use his experiences to highlight possible ways of improvement in PBA. | NA | NA | PBA is a valuable summative and formative assessment tool, with a good level of validity and reliability. Further work is needed to correctly implement PBAs into practice. |
| Sachdeva 2005 | The new paradigm of continuing education in surgery. | Descriptive | NA | NA | The author describes how “continuous professional development integrated with PBLI can help surgeons address their specific learning needs and play a pivotal role in surgeons’ providing the best care to patients.” | NA | NA | “Concerns about traditional CME have resulted in wide acceptance of the concept of CPD, which involves focus on the individual learning needs of physicians across the continuum of their careers. Continuous professional development for surgeons needs to be performance- driven and should be linked to PBLI. The PBLI process involves a cycle of 4 steps—identifying areas for improvement based on self-assessment; engaging in learning; applying new knowledge and skills to practice; and checking for improvement. Continuous professional development and PBLI activities should be documented through the use of portfolios, which can serve as valuable learning and assessment tools. The educational needs of other members of the surgical team, the patients, and patients’ families should also be considered within the context of CPD for surgeons. Thus, a concept of 360° education should be embraced. Continuous professional development activities need to be linked with the overall quality improvement endeavors of the department of surgery and the institution. The skills of educators in- volved with continuing education efforts may need to be enhanced to provide appropriate support for such activities. Relevant and effective CPD that is closely linked to PBLI is key to surgeons’ achieving their individual professional goals and providing the best care to patients.” |
| Lynch et al. 2004 | Assessing practice-based learning and improvement | Descriptive | NA | 4 | This article describes methods used to assess PBLI. | “Six electronic databases were searched using several search terms pertaining to PBLI. The review indicated that 4 assessment methods have been used to assess some or all steps of PBLI: portfolios, projects, patient record and chart review, and performance ratings. Each method is described, examples of application are provided, and validity, reliability, and feasibility characteristics are discussed.” | “This article describes approaches that have been  used to assess PBLI steps. Methods include portfolios,  a project, medical record review, and performance ratings. Each assessment approach has strengths and  weaknesses that have to be weighed against the circumstances in which a method will be used. At the  same time, additional steps can mitigate weaknesses  associated with any single assessment approach. Ultimately, the challenge is one of balancing requirements  for valid and reliable data with the practical limitations  posed by feasibility issues.” | “Portfolios may be the most useful approach to assess residents' PBLI abilities. Active participation in peer-driven performance improvement initiatives may be a valuable approach to confirm practicing physician involvement in PBLI.” |
| Pereira & Dean 2009 | British surgeons' experiences of mandatory online work based assessment | Quantitative | 8.5 | NA | “The study sought to evaluate user satisfaction with the ISCP.” | “A total of 539 users across all surgical specialties (including 122 surgeons acting as assessors) were surveyed in late 2008 by online questionnaire regarding their experiences with the ISCP.” | “Sixty-seven percent had used the tool for at least one year. It was rated above average by only 6% for its registration process and only 11% for recording meetings and objectives. Forty-nine percent described its online assessments as poor or very poor, only 9% considering them good or very good. Seventy-nine percent rated the website's user friendliness as average or worse, as did 72% its peer-assessment tool and 61% its logbook of procedures. Seventy-six percent of respondents had carried out paper assessments due to difficulties using the website. Six percent stated that the ISCP had impacted negatively on their training opportunities, 41% reporting a negative impact overall upon their training; only 6% reported a positive impact. Ninety-four percent did not consider the trainee fee good value, only 2% believing it should be paid by the trainee.” | “The performance of the ISCP leaves large numbers of British surgeons unsatisfied. Its assessments lack appropriate evidence of validity and its introduction has been problematic. With reducing training hours, the increased online bureaucratic burden exacerbates low morale of trainees and trainers, adversely impacting potentially upon both competency and productivity.” |
| Miller & Archer 2010 | Impact of workplace based assessment on doctors' education and performance: a systematic review | Qualitative | NA | 10 | “To investigate the literature for evidence that workplace based assessment affects doctors’ education and performance.” | “Studies of any design that attempted to evaluate either the educational impact of workplace based assessment, or the effect of workplace based assessment on doctors’ performance, were included. Studies were excluded if the sampled population was non-medical or the study was performed with medical students. Review articles, commentaries, and letters were also excluded. The final exclusion criterion was the use of simulated patients or models rather than real life clinical encounters.” | “Sixteen studies were included. Fifteen of these were non-comparative descriptive or observational studies; the other was a randomised controlled trial. Study quality was mixed. Eight studies examined multisource feedback with mixed results; most doctors felt that multisource feedback had educational value, although the evidence for practice change was conflicting. Some junior doctors and surgeons displayed little willingness to change in response to multisource feedback, whereas family physicians might be more prepared to initiate change. Performance changes were more likely to occur when feedback was credible and accurate or when coaching was provided to help subjects identify their strengths and weaknesses. Four studies examined the mini-clinical evaluation exercise, one looked at direct observation of procedural skills, and three were concerned with multiple assessment methods: all these studies reported positive results for the educational impact of workplace based assessment tools. However, there was no objective evidence of improved performance with these tools.” | “Considering the emphasis placed on workplace based assessment as a method of formative performance assessment, there are few published articles exploring its impact on doctors’ education and performance. This review shows that multisource feedback can lead to performance improvement, although individual factors, the context of the feedback, and the presence of facilitation have a profound effect on the response. There is no evidence that alternative workplace based assessment tools (mini-clinical evaluation exercise, direct observation of procedural skills, and case based discussion) lead to improvement in performance, although subjective reports on their educational impact are positive.” |
| Beard 2011 | Workplace-based assessment: the need for continued evaluation and refinement | Descriptive | NA | NA | This article reviews the purpose of WBA and the methods in current use. It also discusses the misuse of WBA and possible solutions, including redesign of the rating scales. | NA | “The primary purpose of WBA is to aid learning by providing trainees with constructive feedback, based on objective, structured assessment (Assessment for Learning).  WBA can be classified as opportunistic (done whenever the opportunity arises) and scheduled (because they require more planning). Opportunistic methods adopted by the ISCP included the mini-Clinical Evaluation Exercise (mini-CEX), Direct Observation of Procedural Skills in Surgery (S-DOPS) and Procedure-Based Assessment (PBA). The scheduled methods include Case-Based Discussion (CBD) and mini-Peer Assessment Tool (mini-PAT).  When WBA was first introduced, minimum numbers of assessments were set as a guide for trainees and assessors, as for the FP. In retrospect, setting minimum numbers was a mistake. Most trainees undertook the minimum, and infrequent assessments led to them being regarded as ‘mini-exams’. Trainees practised informally and only asked for an assessment when they felt confident of achieving a good ‘score’. This also put pressure on the assessors to give a good ‘score’, which they usually did. Thus, the main purpose of WBA as an assessment for learning was lost and it was often regarded as being little more than a tick-box exercise.  The RCP has opted for a different solution for mini-CEX and CBD, using an educationally-referenced scale (e.g. ‘performed at the level expected for Certificate of Completion of Training’). Use of such scales has improved acceptability, validity and reliability, as they are more easily understood by assessors and trainees.” | “Workplace-Based Assessments are a vital part of any competence-based postgraduate curriculum. Whilst it might seem tempting to pick an existing method ‘off the shelf’, utility cannot be assumed as it will depend upon the purpose of the assessment and the culture of the training programme. The purpose, timing and frequency of each assessment require detailed guidance. Continued evaluation and refinement are necessary to ensure that they have good utility and are fit for their intended purpose.” |
| Munsch 2009 | British surgeons' experiences of mandatory online workplace-based assessment - Reply to authors | Descriptive | NA | NA | NA | NA | “In the two years since the launch, the ISCP team has continually been utilizing user feedback, obtained through a wide range of sources, to modify and improve both user interface and the programme utility. Modifications to on-line assessments have been made on the basis of constructive feedback and an active programme of faculty development is helping surgical trainers become familiar with the assessments and other aspects of the ISCP.” | “Constructive criticism by trainees and trainers alike will be heeded by the developers’ and that the ‘versatility and usability of the ISCP will continue to improve’.” |
| Eardley et al. 2011 | The ISCP  systems group: enhancement to the learning agreement and ARCP | Descriptive | NA | NA | Describes changes to learning agreement (LA) and annual review of progression (ARCP) of the Intercollegiate Surgical Curriculum Programme (ISCP) | NA | “The group recommended that it would be more efficient if the PD could select templates that matched the mandatory areas of the syllabus so that essential components could be downloaded en bloc. It should then be more clear which syllabus topics are mandatory and which are placement-specific.”  “It is essential that the whole team demonstrates a collaborative view of the  trainee’s achievements during the clinical placement. Accordingly, the systems group recommended that there should be a mandatory area within the LA’s objective setting section for naming each CS in the faculty team, and that at least one CS must comment on the trainee’s progress in the end-of-placement report. As an aid, an automatic system alert should be sent to the team at completion of the interim review, reminding them to add their commentary.”  “The group felt that more summary detail would be useful and should match the  summary displayed in the ARCP. It is now possible for trainees to upload additional documentation to their portfolios and the group considered that trainees’ reflective writing would be a  useful addition adjacent to their case-based discussions”  “The group recommended that it would be more helpful if the final review were divided into two distinct parts with  guidance about how they should be completed, and that it should be mandatory for at least one CS to contribute to this report. They also felt  that the structure of the form should be consistent with the General Medical Council (GMC)’s Good Medical Practice framework, such that not only clinical skills but other professional skills such as communication skills and academic achievements be recorded.” | .“In summary therefore, these changes should make the process of appraisal and assessment of surgical trainees easier for the trainee, the PD and for the AES. This is however an ongoing process and the ISCP  team welcome suggestions from any  stakeholders of ways in which the functionality of the ISCP website could be improved.” |
| Beard 2008 | Assessment of surgical skills of trainees in the UK | Descriptive | NA | NA | “This article explains some of the new assessments that are being introduced as part of the Intercollegiate Surgical Curriculum Programme (ISCP). This article does not address the issue of selection into surgery. Like examinations, selection is a ‘high-stakes’ assessment which is beyond the scope of this article.” | NA | NA | “Surgical training and assessment in the UK has been criticised in the past for lacking transparency, reliability and validity. The new Intercollegiate Surgical Curriculum Programme (ISCP) has a well-defined, competence-based syllabus and a system of workplace-based assessments and examinations that map to the syllabus. The main aims of workplace-based assessment are to aid learning through objective feedback and to provide evidence that the competencies required to progress to the next level of training have been achieved. Reduction in surgical experience means that more training will need to be undertaken on simulations, although experience and assessment in the operating room must remains the ‘gold-standard’. Simulation training will require the provision of properly resourced surgical skills facilities in every hospital. The key to reliable assessment and constructive feedback is well-trained trainers. Training is a skill that must be learned, and assessment and feedback techniques form part of this. In surgery, it has been assumed that all consultants are trainers but this is clearly not the case. Surgeons will need to follow the example of primary care, where trainers are selected from experienced general practitioners who demonstrate enthusiasm and ability. The reward for the trainer should be protected time for training. The reward for the National Health Service will be better trained surgeons.” |
| Welchman 2012 | Educating the surgeons of the future: the successes, pitfalls and principles of the ISCP | Descriptive | NA | NA | “MMC provided the Royal College of  Surgeons with a mandate to begin a  programme of curriculum redevelopment, known as the Intercollegiate Surgical Curriculum Programme (ISCP). The new curriculum was introduced in 2007 amidst the chaos that accompanied the  roll out of MMC and the Medical Training  Application Service. Consequently, the  frustration felt by surgical trainees during that period has been, in part, attributed to the ISCP. The result is a programme that  suffers from ‘bad press’ and there is little understanding about its principles and ideals. In this review the fundamentals of  the curriculum and the current state of the programme will be considered.” | NA | “What the ISCP has achieved in the six years since its conception should be considered as the greatest transformation of surgical training in modern times. The mandate to begin this project was part of the process of MMC and, as such, trainer and trainee perception of this work has been clouded by anger and dispute over the MMC group’s other activities. Large parts of the new curriculum are sensitive and intelligent responses to public and professional needs and have been very successful. As with all large projects, there are also some areas of concern. These occur throughout the programme and, in the majority, have simple solutions and might be described as ‘teething problems’. The solutions to these problems have been slow to come and some have yet to be considered. Alongside these more minor issues lie areas of significant concern. These include the lack of guidelines on standards for progression, conflicting roles of WBAs and the absence of guidance on adjuncts to experiential teaching. The issues are not insurmountable. If lessons are learnt, particularly on the importance of preparing for change and engagement with the surgical community, the curriculum has potential to continue to develop.  Underpinning all of these issues is the fact that surgical education is worryingly under-researched and lacking in interested educational theorists. This means that there are insufficient data to link educational activities to the real world practice of surgery.” | “Despite these issues, the ISCP has  produced an impressive reform that offers  the surgical community an opportunity to  push the boundaries of surgical education  and, perhaps, lead the world in this field.” |
| Beard et al. 2009 | Workplace based assessment: assessing technical skill throughout the continuum of surgical training | Descriptive | NA | NA | Describes the use of workplace based assessment in surgical training | NA | Workplace based assessments are to aid learning, and are normally trainee led. Trainees are encouraged to use as many different assessors as possible.  PBAs differ from other WBAs, such as DOPS, which use the term ‘satisfactory for that level of training’. This is because PBAs are also intended to be used collectively. At the end of a placement, the collection of PBAs, together with the logbook, will enable the Educational Supervisor or Programme Director to make a summary judgement about the competence of a trainee to perform index procedures to a given standard.  Assessor training is a vital component of WBA, except for peer review, which does not require raters to be trained. | “The main aim of WBA is to aid learning by providing trainees with constructive (formative) feedback. The assessments also help the trainee’s Educational Supervisor (ES) to chart their progress during a placement. Although the principal role of each assessment is formative, the entire collection together with the logbook provide summative evidence for the Programme Director at the Annual Review to help determine whether a trainee has successfully completed a level or module of training.” |
| Brown et al. 2008 | Assessment of operative competency in otolaryngology residency | Quantitative | 8 | NA | “1) Assess current status of operative competency assessment and feedback among US Otolaryngology residency programs. 2) Evaluate correlations between assessment or feedback tools and remediation.” | “The survey was sent to 100 Program Directors inquiring about program size, mode of feedback, assessment tools, surgical portfolios, remediation, and number of residents requiring remediation in past 5 years. Associations between assessment questions and remediation (yes or no) were statistically assessed with the χ2 goodness-of-fit test. For more complicated analyses, multiple logistic regression models were created to assess predictors of the binary outcome of radiation.” | “Among 72 respondents, subjective evaluations are used by 95.8% (69 of 72). Formative feedback after cases is used by 38.9% (28 of 72). Summative feedback at end of the rotation is done by 57.0% (41 of 72). Objective Structured Assessments of Technical Skills is used by 15.3% (11 of 72) of programs, most are large on the basis of number of residents or faculty or both. Operative portfolios are kept by 48.6% (35 of 72) of programs. The percentage of programs with mechanisms for remediation is 41.7% (30 of 72). Similarly, of the programs who responded to the remediation question, the percentage with at least one resident requiring remediation is 37.5%. Programs who report at least one resident remediation are statistically more likely to have a remediation mechanism in place (75% vs. 27.5%; P <.001). There is also a statistically significant association with formative feedback and having resident remediation (58.3% vs. 30%; P = .025).” | “Standardized surgical curricula and assessment tools are needed. Programs with more intensive evaluation or remediation mechanisms are statistically more likely to report residents requiring remediation. This may reflect their ability to better identify the struggling resident. Alternatively, having a resident requiring remediation may be the catalyst that initiates the need for more intensive evaluation tools and remediation mechanisms. The Accreditation Council for Graduate Medical Education's and society will demand that we produce competent surgeons. Therefore, all Otolaryngology training programs should be developing and implementing formal surgical assessment tools, methods to identify residents requiring remediation, and remediation mechanisms.” |
| Brigley et al. 2004 | The educational evaluation of General Professional Practice of Surgery (GPPS). | Descriptive | NA | NA | “In 2003, The Royal College of Surgeons of England piloted, in four NHS Trusts, a new curriculum entitled General Professional practice of surgery (GPPS). This was the product of a working party assigned to write a curriculum to replace the Basic Surgical  Training (BST) manual or ‘Blue Book’ for Senior House Officers  (SHOs).  Two independent evaluations were commissioned. The first, an educational evaluation, reported a favourable response to the introduction of GPPS. It also identified important organisational and resource issues that necessitated a further evaluation of  management issues that affected the curriculum implementation  of GPPS. This paper reports on the educational evaluation.” | NA | “Surgical SHOs were sympathetic towards the values and intentions of GPPS, but acted principally upon those parts of GPPS that would advance their careers. Learning for them had an instrumental value in the competition for SpR posts. SEs too attached credibility to the educational foundations of GPPS, and  had no objection in principle to teaching and assessing under the new curriculum. However, they doubted that its implementation  would be possible until management and resource issues had been addressed. It remained to be seen whether the job planning  process under the new consultant contract would increase protected time for teaching.  Reflective learning and triggered assessments were underdeveloped in this pilot, apparently due to participants’ partial understanding and lack of time to engage with these elements. Induction and curriculum guidance on GPPS for teachers and  learners would be a continuing priority for The Royal College of Surgeons of England. SEs and SHOs acknowledged they had not  fully come to terms with its novel educational concepts and principles. Some SEs suggested the introduction of SE curriculum advisors to promote local educational dialogues. This mission did  not sit well with the demand for a simplified edition of the GPPS document. Attenuated GPPS information would be easier to assimilate, but educationally shallow.  Stakeholders’ views of the GPPS pilot provided diverse  illustrations of a sharpening tension between service and  education. Participants assumed, not unnaturally, that surgical education should be sufficiently flexible to meet career needs and NHS goals. They rightly saw service experience as the fundamental source of SHOs’ learning, but feared that  opportunities for learning and teaching were becoming scarcer. GPPS presents the possibility of enhanced learning from limited  opportunities, but only if all parties are able fully to embrace the educational aims of GPPS and follow through its structured and coherent design for learning.” | The authors conclude that GPPS presents the possibility of enhanced learning from  limited opportunities, but only if all parties are able fully to embrace the educational  aims of GPPS and follow through its structured and coherent design for learning. |
| Mery et al. 2008 | Teaching and assessing the ACGME competencies in surgical residency. | Descriptive | NA | NA | “This article will review the current state of ACGME competencies in general surgery residencies and suggest some methods to teach and evaluate them.” | NA | “Residents must be able to provide patient care  that is compassionate, appropriate, and effective for the treatment of health problems and the promotion of health.  Residents must demonstrate knowledge about established and evolving biomedical, clinical, and cognate (e.g., epidemiological and social-behavioral) sciences and the application of this knowledge to patient care.  Residents must be able to investigate and evaluate  their patient care practices, appraise and assimilate  scientific evidence, and improve their patient care practices.  Residents must be able to demonstrate interpersonal and communication skills that result in  effective information exchange and teaming with  patients, their patients’ families, and professional  associates.  Residents must demonstrate a commitment to  carrying out professional responsibilities, adherence to ethical principles, and sensitivity to diverse  patient population.  Residents must demonstrate an awareness of and  responsiveness to the larger context and system of  health care, as well as the ability to call effectively  on other resources in the system to provide optimal  health care.” | “In the last few years, we have experienced  major changes in resident education in the U.S.—the 80-hour workweek and the ACGME’s six competencies probably being the two most radical. Overall, residency programs are now being required to provide adequate, focused, and relatively equivalent training in less time while documenting residents’ actual learning. The focus of residency learning is thus being shifted from imbibing of knowledge and skills by merely being exposed to the problems in the hospital to a more focused and potentially more meaningful  educational experience. Whether the changes in  the work hours and the implementation of the  set of competencies will accomplish these goals is still to be determined.” |
| Pitts et al. 2005 | Assessment of performance in orthopaedic training. | Descriptive | NA | NA | “In this article, we describe the instruments, their validation and how reliability might be ensured.” | NA | “OCAP consists of two types of instrument: agenda setting/coaching, and assessment.  A cornerstone of OCAP is the collection of performance-based assessments. These are formal, structured assessments of clinical activity (including surgical performance). They take a holistic approach, including understanding a problem, communicating, planning, operating and ensuring clear post-operative instructions.  Performance-based assessments identify and capture relevant activity, which is performed in sufficient numbers often enough to allow reliable measurement. They allow data capture through observations of a series of similar events.   OCAP uses a Delphi method (consensus of an expert group) of validating performance-based assessment.” | “OCAP instruments have been reviewed and triangulation studies indicate they are valid. Early work shows they are reliable and further inter-observer studies are under way. Trainers and trainees have been provided with a structure based on conventional educational instruments and given a means of mapping back to core competencies. The evolving synergy with the orthopaedic log book gives strong supporting evidence of training activity.  We now have a curriculum which meets the standards prescribed by national validating bodies and uses practicable language and taxonomy for trainers and trainees.” |
